# Indoor bacterial load and its correlation to physical indoor air quality parameters in public primary schools

**DOI:** 10.1186/s40248-018-0167-y

**Published:** 2019-01-22

**Authors:** Zewudu Andualem, Zemichael Gizaw, Laekemariam Bogale, Henok Dagne

**Affiliations:** Department of Environmental and Occupational Health and Safety, Institute of Public Health, College of Medicine and Health Sciences, University of Gondar, Gondar, Ethiopia

**Keywords:** Bacterial load, Classrooms, Correlation, Indoor air, Settle plate method

## Abstract

**Background:**

Poor indoor air quality is a great problem in schools due to a high number of students per classroom, insufficient outside air supply, poor construction and maintenance of school buildings. Bacteria in the indoor air environment pose a serious health problem. Determination of bacterial load in the indoor environment is necessary to estimate the health hazard and to create standards for indoor air quality control. This is especially important in such densely populated facilities like schools.

**Methods:**

Institutional based cross-sectional study was conducted among 51 randomly selected classrooms of eight public primary schools from March 29–April 26, 2018. To determine the bacterial load passive air sampling settle plate method was used by exposing a Petri dish of blood agar media for an hour. The Pearson correlation matrix was employed to assess the correlation between bacterial load and physical parameters.

**Results:**

The grand total mean bacterial load was 2826.35 CFU/m^3^ in the morning and 4514.63 CFU/m^3^ in the afternoon. The lowest and highest mean bacterial load was recorded at school 3 (450.67 CFU/m^3^) and school 5 (7740.57 CFU/m^3^) in the morning and afternoon, respectively. In the morning relative humidity (*r* = − 0.7034), PM2.5 (*r* = 0.5723) and PM10 (*r* = 0.6856); in the afternoon temperature (*r* = 0.3838), relative humidity (*r* = − 0.4014) were correlated with indoor bacterial load. *Staphylococcus aureus, Coagulase-negative Staphylococcus species* and *Bacillus species* were among isolated bacteria.

**Conclusions:**

High bacterial load was found in public primary schools in the Gondar city as compared to different indoor air biological standards. Temperature, relative humidity and particulate matter concentration (PM2.5 and PM10) were associated with the indoor bacterial load. *Staphylococcus aureus, Coagulase-negative Staphylococcus species* and *Bacillus species* were among isolated bacterial species. Attention should be given to control those physical factors which favour the growth and multiplication of bacteria in the indoor environment of classrooms to safeguard the health of students and teachers in school.

## Background

Clean air is a basic requirement of life [1]. Most people spend 80–95% of their time in indoor environments by breathing on average 10–14 m^3^ of air per day [2–5]. Millions of children and adults spend 24–30% of their time in a day in school buildings, and they need safe, healthy environments to thrive, learn, and succeed [6, 7].

The indoor air quality has been the object of several studies due to an increasing concern within the scientific community on the effects of indoor air quality upon health, especially as people spend more time indoors than outdoors [8–10]. The quality of air inside, homes, offices, schools or other private and public buildings is an essential determinant of healthy life and people’s well-being [1].

Indoor air pollution is a major problem in people daily life. Efficient corrective methods are urgently needed to combat the problem of indoor air quality: bacteria, pollen grains, smoke, humidity, chemical substances, and gases released by anthropogenic activity which has adverse health effects in humans [11]. Several studies underscore the significant risks of global warming on human health due to increasing levels of air pollution. The last decades have seen a rise in the concentrations of pollens and pollutants in the air. This rise parallels the increase in the number of people presenting with allergic symptoms (e.g., allergic rhinitis, conjunctivitis, and asthma) [12].

Globally, 3.8 million deaths were attributed to indoor air pollution in 2016. More than 90% of air pollution-related deaths occur in low- and middle-income countries, mainly in Asia and Africa, followed by low- and middle-income countries of the Eastern Mediterranean region, Europe, and Americas [13]. Bioaerosols contribute about 5–34% of indoor air pollution [5, 6, 14, 15].

Indoor air quality problems in schools may be even more serious than in other categories of buildings, due to higher occupant density, poor sanitation of classrooms, and insufficient outside air supply, aggravated by frequent poor construction and maintenance of school buildings [16]. Poor indoor air quality can also affect scholarly performance and attendance since children are more vulnerable than adults to health risks from exposure to the environmental hazard [16–18].

Therefore, the purpose of this research was to assess the bacterial quality of indoor air in public primary schools to increase awareness and provide references for better understanding about bacterial indoor air quality problems in public primary schools.

## Methods and materials

### Study design and study area

Institutional based cross-sectional study was conducted to assess indoor bacterial load and its relation to physical indoor air quality parameters of public primary schools in Gondar city, Northwest Ethiopia. Gondar city is located in the northern part of Ethiopia in Amhara national regional state, North Gondar zone at a distance of 727 km from Addis Ababa and 173.09 km from the regional capital Bahir Dar at the 12°45’north latitude and 37 ° 45′ east longitudes. In Gondar city there are twenty public primary schools from grade 1–8 with a total of 27,766 students enrolled in 266 classrooms [19].

### Sample size and sampling procedures

The sample size was determined based on environmental sampling and sample size determination methods [20]. Manly formula was used to determine sample size [21] by using the following equation.

$$ n=\frac{4{\upsigma}^2\ }{\updelta^2} $$

Where *n* = Number of samples, σ = Standard deviation, δ = Acceptable error [δ is half of the width of a 95% the confidence interval on the mean ($\overline{X}\pm$δ)].

From a total of twenty public primary schools, 40% of the schools were selected through simple random sampling and 20% of classrooms at schools were selected as study unit through simple random sampling.

The mean and standard deviation (σ) of eight randomly selected public primary schools was 13.3 and 5.37 respectively, by taking 3% acceptable error (δ).

$$ n=\left[\frac{4\ast {(5.37)}^2}{(1.5)^2}\right]=51 $$

A total of fifty-one classrooms were selected from eight public primary schools of Gondar city by simple random sampling technique.

### Air sampling procedures

Air samples were taken from 51 randomly selected classrooms from eight public primary schools in Gondar city. Bacterial measurements were made by passive air sampling method i.e., the settle plate method. Standard Petri dishes with 9 cm diameter (63.585 cm^2^ areas) containing culture media were exposed. Bacterial contamination determination was based on the count of the microbial fall out on to Petri dishes left open to the air, according to the 1/1/1 scheme (for 1 h, 1 m away from the floor, at least 1 m away from walls or any obstacle) [14]. Bacteria were collected on blood agar media to which an antifungal agent (Griseofulvin) had been added to inhibit the growth of fungi. To determine the bacterial load with respect to environmental variation, sampling was done in the morning (at 6:30 am before students enter to the classroom) and afternoon (5:00 pm, after students left the classroom). After exposure, the sample was taken to the laboratory (Department of Biology, at the University of Gondar) and incubated at 37 °C for 24 to 48 h. Colony forming units (CFU) was enumerated, CFU/m^3^ microbial concentration was determined, using the following equation [22].

$$ \raisebox{1ex}{$\mathrm{N}=\mathrm{a}\ast 10000$}\!\left/ \!\raisebox{-1ex}{$\mathrm{bt}\ast 0.2$}\right. $$

Where N = Microbial CFU/m^3^ of indoor air; a = Number of colonies per Petri dish; b = Dish surface area (cm^2^); t = Exposure time.

Individual bacterial isolates were identified using standard methods (including colonial morphology, microscopy, and biochemical tests) [23, 24].

Parallel with bacterial sample collection, data on physical parameters such as CO_2_ concentration, particulate matter concentration (PM2.5 and PM10), indoor temperature, and relative humidity were measured by Aireveda. To minimize dilution of air contaminants, openings like doors and windows were closed [7, 25, 26]. In addition, the movement of people during sampling was restricted to avoid air disturbance and newly emitted microorganisms.

### Data analysis

Statistical analyses were carried out using STATA/SE 14.0. To assess the correlation of bacteria concentration with environmental factors like carbon dioxide concentration, particulate matter concentration (PM2.5 and PM10), temperature and relative humidity Pearson correlation was employed. One way analysis of variance (ANOVA) was carried out to know the mean difference of the bacterial load in public primary schools.

## Results

### Bacterial load

The concentrations of bacterial aerosols in the indoor environment of public primary schools in Gondar city, estimated with the use of the settle plate method, the lowest and highest bacterial load was estimated in the morning in school 1 (208 CFU/m^3^) and in the afternoon in school 5 (23,504 CFU/m^3^) (Table 1).

**Table 1:** Statistical summary of bacterial load, in public primary schools of Gondar city, Northwest Ethiopia, 2018. (*n* = 51)

Name of School	Number of classrooms	Mean	Standard deviation	Minimum	Maximum	Median
Morning bacterial load						
School 1	5	702.00	316.44	390	1183	741
School 2	7	1131.00	515.87	559	2145	1066
School 3	3	450.67	314.52	208	806	338
School 4	8	5843.50	1973.45	3588	8528	5356
School 5	7	5588.14	2167.85	3341	9100	4420
School 6	8	3284.13	1185.78	2067	5668	3321.5
School 7	7	748.43	179.95	585	1105	767
School 8	6	2331.33	801.49	1430	3666	2158
Total	51	2826.35	2408.76	208	9100	2093
Afternoon bacterial load						
School 1	5	1510.60	1267.53	728	3757	1001
School 2	7	5404.29	2320.31	2301	8502	5460
School 3	3	507.00	144.76	351	637	533
School 4	8	5073.25	2517.63	2600	9984	4082
School 5	7	7740.57	7228.03	2340	23,504	5304
School 6	8	1158.63	709.06	260	2652	994.5
School 7	7	4561.57	2031.61	819	7332	4332
School 8	6	7895.33	1403.10	5720	9880	7943
Total	51	4514.63	3918.20	260	23,504	3939

The grand total mean bacterial load was 2826.35 and 4514.63 CFU/m^3^ in the morning and afternoon, respectively, while the overall mean bacterial load was 3670.49 CFU/m^3^ (Table 1). ANOVA test result was presented to show the mean bacterial load difference among different public primary schools. The test showed that there was a significant mean bacterial load difference among public primary schools at *p* < 0.001 (Table 2).

**Table 2:** ANOVA test result on mean bacterial load difference among public primary schools of Gondar city, Northwest Ethiopia 2018

Analysis of Variance					
Source variation	Sum of Square	Degree of freedom	Mean Square	F	Sig.
Between groups	189,395,110	7	27,056,444.3	7.67	< 0.001
Within groups	151,708,969	43	3,528,115.56		
Total	341,104,079	50	6,822,081.58		

### Physical parameters of indoor air environments

During physical parameter measurement, it was found that all examined classrooms did not have an HVAC (heating, ventilation, and air conditioning) system. The ranges of carbon dioxide concentration, indoor temperature, relative humidity, and particulate matter concentration (PM2.5 & PM10) during sampling time ranged from 401 to 550 ppm, 12 to 24 °C, 14 to 64%, 7 to 173 μg/m^3^, and 21 to 277 μg/m^3^ respectively (Table 3).

**Table 3:** Statistical summary of physical indoor air quality parameters in public primary schools of Gondar city, Northwest Ethiopia, 2018 (*n* = 51)

Variables	Mean	Standard Deviation	Median	Minimum	Maximum
CO_2_ 6:30 am	458.54	30.44	453	406	550
CO_2_ 5:00 pm	429.12	27.05	422	401	548
T ^°^*C* 6:30 am	14.27	0.94	14	12	16
T ^°^*C* 5:00 pm	19.02	3.08	20	15	24
RH%6:30 am	39.37	14.25	45	21	61
RH%5:00 pm	36.37	13.89	39	14	57
PM2.5 6:30 am	68.18	43.42	48	18	173
PM2.5 5:00 pm	12.82	3.38	13	7	22
PM10 6:30 am	138.06	68.64	129	42	277
PM10 5:00 pm	55.92	22.06	51	21	144

*N.B* Carbon dioxide, (CO_2_, ppm); Temperature, (T, °C); Relative humidity, RH (%); concentration of particulate matter concentration PM10 and PM2.5 (μg/m^3^)

### Isolated bacterial species

Three bacterial species were isolated; *Bacillus species, Staphylococcus aureus* and C*oagulase-negative Staphylococcus (CoNS*) species. *Bacillus species* was found in all public primary schools (Table 4).

**Table 4:** Type of microorganism isolated from each public Primary schools in Gondar city Northwest Ethiopia, 2018 (*n* = 51)

School ID	Number of classrooms	Bacterial Isolates		
		SA	BA	CoNS
School 1	5	**–**	**+**	**+**
School 2	7	**+**	**+**	**+**
School 3	3	**+**	**+**	**+**
School 4	8	**+**	**+**	**+**
School 5	7	**+**	**+**	**–**
School 6	8	**+**	**+**	**+**
School 7	7	**+**	**+**	**+**
School 8	6	**+**	**+**	**–**
Total	51	7	8	6

*N.B.* Staphylococcus aureus *(SA),* Bacillus species *(BA),* Coagulase negative staphylococcus species (*CoNS*), **+** = Present, **−** = absent

According to the European sanitary standards for non-industrial premises, the degree of air pollution by bacteria population across the various classrooms of the eight public primary schools ranges largely between high to very high (Table 5).

**Table 5:** Assessments of bacterial indoor air quality in the selected eight public Primary schools in Gondar city, according to the sanitary standards for non-industrial premises (*n* = 51)

		Bacterial									
Sampling time		6:30–7:30 am					5:00–6:00 pm				
Range of values (CFU/m^3^)		< 50	50–100	100–500	500–2000	> 2000	< 50	50–100	100–500	500–2000	> 2000
Degree of Air Pollution		Very low	Low	Intermediate	High	Very high	Very low	Low	Intermediate	High	Very high
School	School 1	**–**	**–**	**–**	**√**	**–**	**–**	**–**	**–**	**√**	**–**
	School 2	**–**	**–**	**–**	**√**	**–**	**–**	**–**	**–**	**–**	**√**
	School 3	**–**	**–**	**√**	**–**	**–**	**–**	**–**	**–**	**√**	**–**
	School 4	**–**	**–**	**–**	**–**	**√**	**–**	**–**	**–**	**–**	**√**
	School 5	**–**	**–**	**–**	**–**	**√**	**–**	**–**	**–**	**–**	**√**
	School 6	**–**	**–**	**–**	**–**	**√**	**–**	**–**	**–**	**√**	**–**
	School 7	**–**	**–**	**–**	**√**	**–**	**–**	**–**	**–**	**–**	**√**
	School 8	**–**	**–**	**–**	**–**	**√**	**–**	**–**	**–**	**–**	**√**

*NB*: ≤ 500 CFU/m^3^ is the permissive standard, (**√**) in the range; (**−**) not in the range

Relative humidity, particulate matter concentration and temperature correlated with an indoor bacterial load of public primary schools from all physical indoor air quality parameters; relative humidity had a negative strong correlation with indoor bacterial load (Table 6).

**Table 6:** Pearson correlation coefficients between indoor bacterial concentration & physical indoor air quality parameters in public primary schools of Gondar city, Northwest Ethiopia, 2018. (*n* = 51)

Variables	Bacteria	CO_2_	T (^°^*C*)	RH (%)	PM2.5	PM10
Morning						
Bacteria	1.00					
CO_2_	0.1049	1.00				
T ^°^*C*	0.0007	−0.0928	1.00			
RH%	−0.7034^a^	0.2237	−0.0839	1.00		
PM2.5	0.5723^a^	0.2226	−0.0767	− 0.3634^a^	1.00	
PM10	0.6856^a^	0.1573	−0.0762	−0.5493^a^	0.9447^a^	1.00
Afternoon						
Bacteria	1.00					
CO_2_	−0.1641	1.00				
T ^°^*C*	0.3838^a^	−0.1820	1.00			
RH%	−0.4014^a^	0.4510^a^	−0.8420^a^	1.00		
PM2.5	−0.1100	0.6676^a^	−0.2036	0.4368^a^	1.00	
PM10	0.1856	0.5418^a^	0.2903^a^	−0.0771	0.4460^a^	1.00

N.B = ^a^ Correlation is significant at the 0.05 level; Carbon dioxide, (ppm); Temperature, T (°C); Relative humidity, RH (%); concentration of particulate matter PM10 and PM2.5 (μg/m^3^)

## Discussion

The bacterial load of indoor air environments of public primary schools in Gondar city was found in the range between 208 and 23,504 CFU/m^3^ with a mean bacterial load of 3670.49 CFU/m^3^, the finding of this study was higher than the findings of other studies, one conducted in Poland, [27] and another in Malaysia, [28].

There are no generally accepted threshold limit values concerning concentrations of the air of indoor bacteria, and the obtained results could be compared only with the values recommended by various authors or institutions. The work conducted by a WHO expert group on assessment of health risks of biological agents in indoor environments suggested that total microbial concentration should not exceed 1000 CFU/m^3^ [29], whereas other scholars considered that 750 CFU/m^3^ should be the limit for bacteria [30]. Airborne microbial concentrations ranging from 4500 to 10,000 CFU/m^3^ also have been suggested as the upper limit for ubiquitous bacterial aerosols [31]. According to the sanitary standards of the European Commission for non-industrial premises, the permissible limits of bacterial load were ≤ 500 CFU/m^3^ [32]. The variation of bacterial load in indoor environments might be due to environmental factors such as ventilation system of classroom, temperature, humidity, and particulate matter concentration.

The finding of isolated bacterial species of the present study partly agrees with the work by Hussin N. et al. [28]. Likewise, it was harmonized in a study conducted in India [33], and it was partly agreed in the work by Naruka K. et al., [34].

In this study, the temperature of the indoor environment had a positive correlation with total airborne bacteria in the afternoon (*r* = 0.3838) while there was no correlation in the morning airborne bacterial concentration. During the study, the temperature ranged from 12 to 16 °C and 15–26 °C in morning and afternoon, respectively. This was consistent with the results reported by Brągoszewska Ewa, et al. [35], but inconsistent with the results reported by Naruka et al. [34], where the temperature was negatively correlated, and Hayleeyesus S. et al. [14] where there was no correlation between temperature and indoor bacterial load.

The bacterial load would be significantly correlated with indoor temperature, i.e., the concentration of aerosols will increase as the temperature increases [35], but the variation might be due to the fact that other environmental factors increase the concentration of bacteria in classrooms and the number of students may result in a great diversity of high bacterial load [36].

In this study relative moderate to strong humidity was negatively correlated with total airborne bacteria in the afternoon (*r* = − 0.4014) and in the morning (*r* = − 0.7034), respectively. The RH in public primary schools ranged from 21 to 62 % and 14–57 % in morning and afternoon, respectively. The negative correlation between relative humidity and indoor airborne bacterial load was not consistent with what is expected since a strict correlation between bacterial load and relative humidity was already reported by Brągoszewska Ewa, et al. [35], Huang H, et al. [37]. The possible explanation might be that if relative humidity decreases, bacterial load becomes decreased because the viability of aerosols becomes inhibited if relative humidity is too low, because a dry environment decreases the metabolism and physiological activities of microorganisms [35].

Correlation of PM2.5 was positively strong with total airborne bacteria (*r* = 0.5723), while there is no correlation in the afternoon airborne bacterial concentration. The positive correlation of this finding is in agreement with a study conducted in Poland [38], but in other study conducted in Poland [35], PM2.5 was negatively correlated.

In this study, PM10 had a strong positive correlation with airborne bacterial load (*r* = 0.6856) but there was no correlation with the afternoon airborne bacterial load. The positive correlation is supported by a study conducted in Poland [35]. The possible explanation might be due to the fact that the PM10 increases the bacterial load that increases because of bioaerosols attached to coarse solid particles [39]; whereas the afternoon particulate matter concentration was not correlated with indoor bacterial load due to other environmental factors which have a more significant correlation with concentration bacterial load as compared to PM10, and the concentration of PM10 in the afternoon is lower when compared in the morning.

## Conclusions

A high bacterial load was found in public primary school classrooms in the Gondar city as compared with different indoor air biological standards. Temperature, humidity, and particulate matter concentration (PM2.5 and PM10) were associated with the indoor bacterial load. *Staphylococcus aureus, Coagulase-negative Staphylococcus, and* B*acillus* were among isolated bacterial species. Attention should be given to control those physical factors which favour the growth and multiplication of bacteria in the indoor environment of classrooms to safeguard the health of students and teachers in school.

## Abbreviations

°C: Degree centigrade
am: Ante meridian
ANOVA: Analysis of variance
CFU: Colony forming units
cm^2^: Centimeter square
m^3^: Cubic meter
PM: Particulate matter
pm: Post meridian
ppm: Part per million
RH: Relative humidity
WHO: World Health Organization.
μg: Microgram
